# Clinical therapeutic effects of high-flow nasal oxygen therapy in patients with acute exacerbation of chronic obstructive pulmonary disease: A protocol for systematic review and meta-analysis

**DOI:** 10.1097/MD.0000000000024084

**Published:** 2021-03-26

**Authors:** Xu-Chi Chen, Chang Liu, Shi-Jun Ma, Dong-Dong Yan, Shuai Wang, Jian Dai

**Affiliations:** a Intensive Care Unit, Wuchang Hospital of Wuhan; b Intensive Care Unit, Wuchang Hospital Affiliated to Wuhan University of Science and Technology, Wuhan, Hubei, China.

**Keywords:** a systematic review, chronic obstructive pulmonary disease, effectiveness, high-flow oxygen

## Abstract

**Background:**

For patients with acute exacerbation of chronic obstructive pulmonary disease (AECOPD) complicated by respiratory acidosis, noninvasive ventilation therapy is thought to be the first-line treatment. In patients with AECOPD, the effect of high-flow nasal oxygen therapy is not well studied. In this study, the existing data will be synthesized to obtain an effective rate of movement of nasal oxygen therapy in patients with AECOPD.

**Methods:**

Using PubMed, EMBASE, Cochrane Library, Web of Science, Scopus, a systematic search will be undertaken to identify randomized controlled trails (RCTs) on the clinical therapeutic effects of rate of movement of nasal oxygen therapy in patients with AECOPD without language constraints from their onset to November 2020. To classify potentially qualifying tests, we will also review Google Scholar, ClinicalTrials.gov, and the reference lists of included studies. Two independent reviewers will review inclusion trials and execute data extraction. Research bias and quality will be measured using the Cochrane Collaboration Bias Method 2.0. The findings of the analysis will be pooled using a formula of fixed-effects or random-effects. We will address any dispute by dialogue, and cases of disagreement will be mediated by a third author.

**Results:**

The current research will examine the clinical therapeutic results of patients with AECOPD with rate of movement of nasal oxygen therapy.

**Conclusion:**

To assess the efficacy of rate of movement of nasal oxygen therapy in patients with AECOPD, the present analysis would provide consistent facts.

**OSF registration number:**

November 18, 2020.osf.io/umd48. (https://osf.io/umd48/).

## 1 Introduction

One of the most common causes of compromised health is a chronic obstructive pulmonary disease (COPD).^[1]^ Based on 2015 projected Global Burden of Disease, COPD has affected about 174.5 million cases.^[2]^ The estimated probability of developing COPD by the age of 80 years was estimated to be 28 per cent based on population-level health administrative data published in 2011.^[3]^ Acute exacerbations of COPD (AECOPD) are characterized by an acute deterioration of respiratory symptoms involving the use of treatment and by variable clinical signs and causative factors.^[4–6]^ These are incidents of considerable significance in the course of the illness, with an important burden on the quality of health, an increased need for hospitalization, a reduction in lung capacity, and an increased risk of morbidity and mortality.^[4,7]^

AECOPD can be induced by many factors, with respiratory infections caused by bacteria or viruses and environmental factors, such as contamination or allergens, being the most common causes. Hospitalization or emergency room admissions may be needed for multiple exacerbations and may be associated with acute respiratory failure. Rate of movement of nasal oxygen therapy is currently a common respiratory support system in patients with COPD. rate of movement of nasal oxygen therapy decreases the respiratory rate and increases oxygenation in acute hypoxic respiratory failure.^[8]^ In adult patients with acute respiratory failure, Zhao et al found that rate of movement of nasal oxygen therapy was not equivalent to traditional oxygen therapy but not to noninvasive mechanical ventilation in the avoidance of intubation.^[9]^ Several studies have also found rate of movement of nasal oxygen therapy to be more relaxed and minimize dyspnea greater than traditional oxygen therapy or noninvasive mechanical ventilation.^[10–13]^ The ultimate purpose of the present research was therefore to summarize available data investigating the efficacy of rate of movement of nasal oxygen therapy in patients with AECOPD.

## 2 Methods

This protocol will be published in compliance with the recommendations for the Preferred Reporting Items for Systematic Analysis and Meta-Analyses Protocols (PRISMA-P).^[14]^ This protocol has been registered on the Open Science Framework (OSF, http://osf.io/), and the registration DOI number is 10.17605/OSF.IO/UMD48.

## 3 Eligibility criteria

### 3.1 Types of studies

Only randomized controlled trails (RCTs) will be used for the clinical therapeutic benefits of rate of movement of nasal oxygen therapy in AECOPD patients.

### 3.2 Types of participants

The participants were patients with a clinically confirmed diagnosis of AECOPD.

### 3.3 Types of interventions and comparisons

Compared to traditional oxygen therapy, noninvasive mechanical ventilation or no care, we will use rate of movement of nasal oxygen therapy RCTs as a single intervention or in conjunction with another therapy process.

### 3.4 Types of outcome measures

Respiratory rates, death, and duration of stay in the intensive care unit were the main effects. Partial arterial oxygen pressure, partial arterial blood carbon dioxide pressure, forced expiratory volume in the first second, reintubation rate, and oxygenation index were the minor outcomes.

## 4 Search methods for primary studies

### 4.1 Electronic searches

Using PubMed, EMBASE, Cochrane Library, Web of Science, Scopus, a systematic search will be undertaken to identify randomized controlled trails (RCTs) on the clinical therapeutic effects of rate of movement of nasal oxygen therapy in patients with AECOPD without language constraints from their onset to November 2020. The search strategy for PubMed is shown in Table 1.

**Table 1:** The search strategy for PubMed.

Number	Search terms
1	high-flow therapy
2	high-flow nasal oxygen
3	high-flow nasal therapy
4	high-flow nasal cannula
5	HFNC
6	1 or 2–5
7	“Pulmonary Disease, Chronic Obstructive”[Mesh]
8	COPD
9	7 or 8
10	Randomized Controlled Trial [Publication Type]
11	randomized controlled trial
12	randomized
13	10 or 11–12
14	6 and 9 and 13

### 4.2 Searching other sources

To classify potentially qualifying tests, we will also review Google Scholar, ClinicalTrials.gov, and the reference lists of included studies.

## 5 Data collection and analysis

### 5.1 Selection of studies

**Figure 1:**
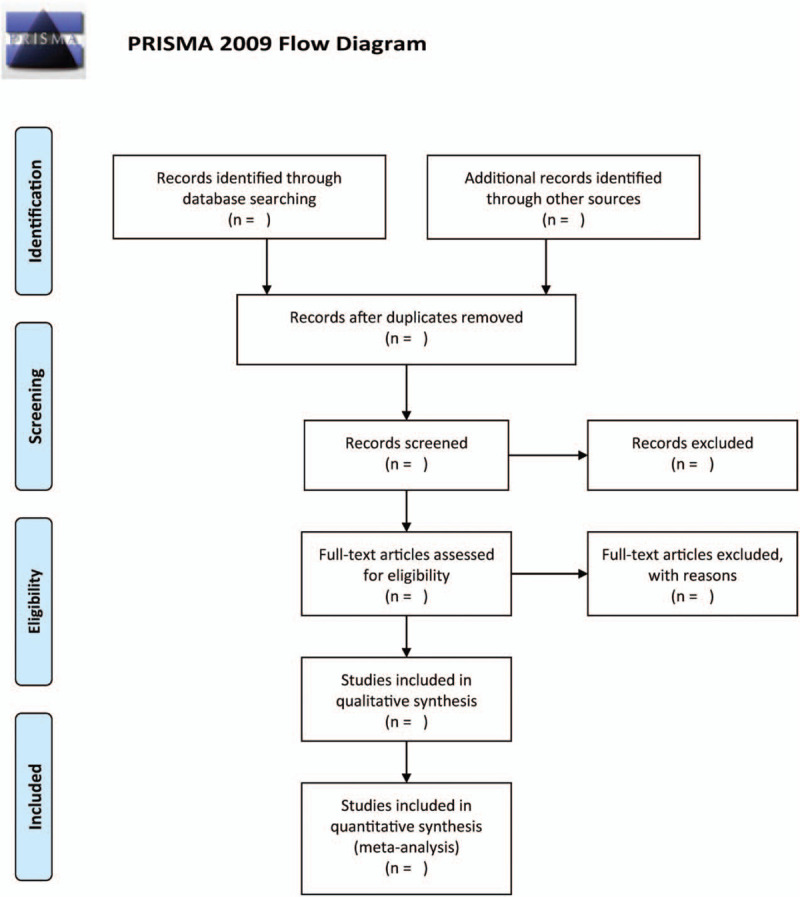
The research flowchart.

The literature will be separately screened by 2 reviewers. First, through filtering titles and abstracts, they removed duplicated and nonRCT studies. Second, to access qualified research, they study the full text. We will address any dispute by dialogue, and cases of disagreement will be mediated by a third author. The flow chart is shown in Figure 1.

### 5.2 Data extraction

Using a pre-designed data extraction form, 2 reviewers will extract data from the included studies separately. Publication details, research eligibility requirements, study specifics, participant attributes, intervention definition and reference, and outcome measures may be included in the derived material. We will address any dispute by dialogue, and cases of disagreement will be mediated by a third author.

### 5.3 Risk of bias assessment

The probability of bias in the included research will be independently assessed by 2 reviewers based on the methods defined in the Cochrane Collaboration's Risk of Bias Tool 2.0. We will address any dispute by dialogue, and cases of disagreement will be mediated by a third author. Centred on the following domains, studies will be evaluated: Random sequence generation and distribution concealment (selection bias), sample and staff blindness (performance bias), inadequate result reports (attrition bias), blinding (detection bias), biased outcome monitoring (reporting bias), and other bias outlets. If the included analysis meets the above criteria entirely, it shows that the risk of bias is low and the quality of the literature is grade A; slightly meets the above criteria, it shows that the risk of bias is modest and the quality of the literature is grade B; if the above criteria are not met at all it shows that the risk of bias is strong and the degree of quality of the literature is C.

### 5.4 Measures of treatment effect

Dichotomous data together with 95% confidence intervals will be expressed as the risk ratio. Continuous data would be expressed as the mean difference or standardized mean difference along with 95% CI.

### 5.5 Management of missing data

If information is incomplete, we will contact the relevant author to retrieve the missing information. We would review records of studies with missing data and disclose the explanations for missing data if we fail to retrieve adequate data.

### 5.6 Assessment of heterogeneity

An *I*^*2*^ metric will measure statistical heterogeneity. We expect to consider a heterogeneity level of more than 50 per cent as major heterogeneity, and the data will be pooled using a model of random effects.

### 5.7 Sensitivity analysis

We will conduct a sensitivity analysis using suitable techniques to determine the reliability of the findings if we find adequate studies.

## 6 Discussion

Rate of movement of nasal oxygen therapy has increasingly been commonly used in patients with AECOPD. The efficacy of treatment with rate of movement of nasal oxygen in patients with AECOPD, however, remains inconclusive. To assess the efficacy of rate of movement of nasal oxygen therapy in patients with AECOPD, we will therefore perform the present review. We hope these results will provide physicians with the framework for AECOPD's high-flow nasal oxygen therapy and provide the best alternative for patient therapy.

## Author contributions

**Conceptualization:** Xuchi Chen, Dongdong Yan, Shuai Wang.

**Data curation:** Chang Liu, Shijun Ma, Dongdong Yan, Jian Dai.

**Formal analysis:** Xuchi Chen, Jian Dai.

**Funding acquisition:** Chang Liu, Shijun Ma, Dongdong Yan.

**Investigation:** Chang Liu.

**Methodology:** Shijun Ma, Shuai Wang, Jian Dai.

**Project administration:** Chang Liu, Dongdong Yan.

**Resources:** Shijun Ma, Dongdong Yan.

**Software:** Xuchi Chen, Shuai Wang.

**Supervision:** Chang Liu.

**Validation:** Xuchi Chen, Shijun Ma, Dongdong Yan, Jian Dai.

**Visualization:** Shijun Ma, Shuai Wang.

**Writing – original draft:** Xuchi Chen, Chang Liu, Jian Dai.

**Writing – review & editing:** Xuchi Chen, Jian Dai.
